# YAP Inhibition Alleviates Simulated Microgravity-Induced Mesenchymal Stem Cell Senescence via Targeting Mitochondrial Dysfunction

**DOI:** 10.3390/antiox12050990

**Published:** 2023-04-24

**Authors:** Wenjun Lv, Xiufen Peng, Yun Tu, Yisong Shi, Guanbin Song, Qing Luo

**Affiliations:** Key Laboratory of Biorheological Science and Technology, Ministry of Education, College of Bioengineering, Chongqing University, Chongqing 400044, China; lvwenjun@cqu.edu.cn (W.L.); 202119021067@cqu.edu.cn (X.P.); tuyun@cqu.edu.cn (Y.T.); shiyis@cqu.edu.cn (Y.S.)

**Keywords:** simulated microgravity, mesenchymal stem cells, cell senescence, YAP, mitochondrial dysfunction

## Abstract

Weightlessness in space leads to bone loss, muscle atrophy, and impaired immune defense in astronauts. Mesenchymal stem cells (MSCs) play crucial roles in maintaining the homeostasis and function of the tissue. However, how microgravity affects the characteristics MSCs and the related roles in the pathophysiological changes in astronauts remain barely known. Here we used a 2D-clinostat device to simulate microgravity. Senescence-associated-β-galactosidase (SA-β-gal) staining and the expression of senescent markers p16, p21, and p53 were used to evaluate the senescence of MSCs. Mitochondrial membrane potential (mΔΨm), reactive oxygen species (ROS) production, and ATP production were used to evaluate mitochondrial function. Western blot and immunofluorescence staining were used to investigate the expression and localization of Yes-associated protein (YAP). We found that simulated microgravity (SMG) induced MSC senescence and mitochondrial dysfunction. Mito-TEMPO (MT), a mitochondrial antioxidant, restored mitochondrial function and reversed MSC senescence induced by SMG, suggesting that mitochondrial dysfunction mediates SMG-induced MSC senescence. Further, it was found that SMG promoted YAP expression and its nuclear translocation in MSCs. Verteporfin (VP), an inhibitor of YAP, restored SMG-induced mitochondrial dysfunction and senescence in MSCs by inhibiting YAP expression and nuclear localization. These findings suggest that YAP inhibition alleviates SMG-induced MSC senescence via targeting mitochondrial dysfunction, and YAP may be a potential therapeutic target for the treatment of weightlessness-related cell senescence and aging.

## 1. Introduction

Cellular senescence is influenced by various mechanical forces, including shear stress, stretching, and tension [1,2,3]. The space environment has the characteristics of strong radiation, ultra-low temperature, and microgravity, among which microgravity is the main mechanical force affecting the health of astronauts [4,5]. So far, studies on the effect of microgravity on cell senescence are limited to erythrocytes, skeletal muscle myoblasts, endothelial cells, and pheochromocytoma cells (PC12) [6,7,8,9], and the effect of microgravity on cell senescence and the related mechanism still is barely known.

Mesenchymal stem cells (MSCs) are adult stem cells with the capability of self-renewal differentiation and paracrine, which facilitate tissue renewal and regeneration and thereby maintain homeostasis and function of tissues [10,11]. However, these capabilities were dramatically reduced with the senescence of MSCs [12]. Studies have demonstrated that outer space environments lead to significant health problems for astronauts, such as bone loss, muscle atrophy, and cardiovascular diseases [13,14,15]. In normal gravity (NG), studies have found that MSC senescence impairs their ability to proliferate and differentiate, thereby affecting bone repair [16,17]. Other studies found that aging mice with MSC mitochondrial dysfunction leads to skeletal muscle atrophy and myofiber loss [18]. In addition, the senescence of MSCs displayed reduced immunosuppressive effects [19]. These findings indicate that the pathological changes in astronauts in space may be associated with the senescence of MSCs. However, the effect of microgravity on MSC senescence is unknown. Studies on the effect of microgravity on MSC senescence is conducive to understanding the role of MSC senescence in the development of physiological and pathological changes in astronaut and can provide new ideas and methods for long-term space life to maintain health.

Yes-associated protein (YAP) is a crucial effector in the Hippo signaling pathway regulating development, homeostasis, and regeneration [20,21]. When activated, YAP translocates into the nucleus, binds to transcription factors, and induces transcription of target genes, which regulates cell behaviors [22,23]. Recently, accumulating studies have shown that YAP can regulate cell senescence by affecting ATM (ataxia-telangiectasia mutated), p53-p21, p16-CDK-RB, autophagy, AMPK, mTOR, and SIRT1 signaling pathways [24,25,26,27,28]. However, the role of YAP in regulating cell senescence varies according to cell type. In human colon cancer cells (HCT116), endothelial cells, and vascular tissue, the upregulation of YAP accelerates senescence [29,30]. Nevertheless, in chondrocytes, astrocytes, and glioblastoma cells, YAP downregulation promotes senescence [31,32]. Oroxylin A, an anticarcinogen, upregulated YAP to reduce the senescence of ethanol-treated normal human liver cells (L02) [33]. On the contrary, the addition of YAP inhibitor verteporfin (VP) increased the cell senescence of human periodontal ligament stem cells [34]. However, whether YAP regulates MSC senescence under microgravity is unclear.

In this study, we aimed to investigate the effect of simulated microgravity (SMG) on MSC senescence and the role of YAP and mitochondrial dysfunction in this process. We found that YAP inhibitor VP alleviated SMG-induced MSC senescence by targeting mitochondrial dysfunction. These findings indicate the crucial role of YAP in SMG-induced MSC senescence, which may further illuminate our understanding of how the microgravity environment affects cell senescence.

## 2. Materials and Methods

### 2.1. Simulated Microgravity

**Figure 1:**
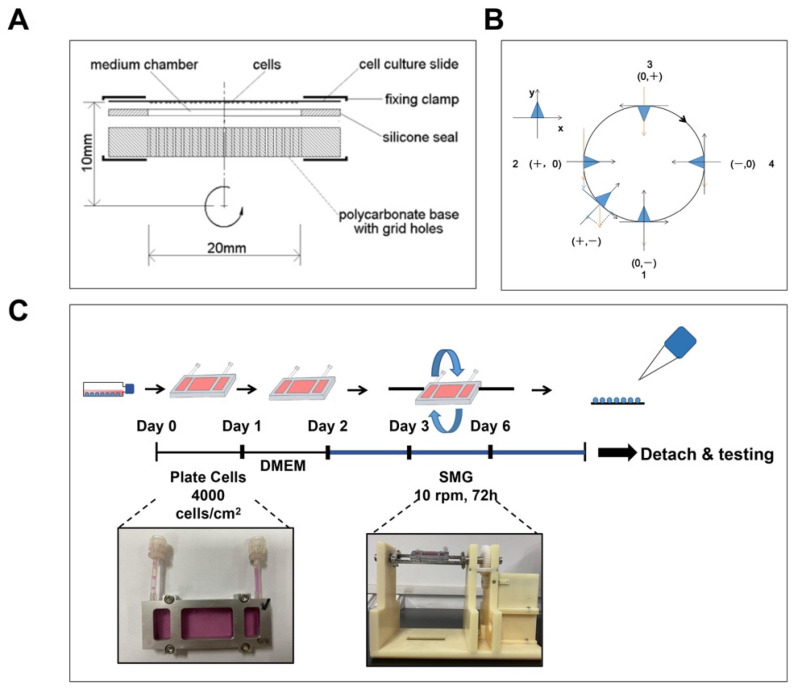
Experimental design and setup for SMG treatment. (**A**) Illustration of a fixed chamber of the 2D-clinostat device. (**B**) The principle of SMG in this experiment. (**C**) Experimental design for this study. MSCs were cultured in a fixed chamber of the 2D-clinostat device to achieve SMG. For NG, MSCs were cultured in a chamber without rotation. MSCs—mesenchymal stem cells; NG—normal gravity; SMG—simulated microgravity.

The 2D-clinostat device was used to simulate the microgravity environment of in vitro culture, which was provided by the National Microgravity Laboratory, Institute of Mechanics, Chinese Academy of Sciences (Beijing, China). The MSCs were seeded at a density of 4000 cells/cm^2^ in a chamber and left to adhere for 24 h, and the MSCs were starved with DMEM serum-free medium for 24 h. The MSCs were then treated with a 2D-clinostat device to simulate microgravity and collected from the device after 24 h, 48 h, and 72 h for various experiments (Figure 1C).

### 2.2. MSC Isolation and Culture

All animal experiments and experimental procedures were conducted following international laboratory animal welfare regulations and the requirements of the Ethics Committee of Chongqing University (CQU-IACUC-RE-202205-001). Male Sprague Dawley (SD) rats with weights in the range of 50–60 g were selected, and the hind limb bones were separated on the ultra-clean bench. The bone marrow was flushed out from the marrow cavity using a syringe and incubated in a humidified incubator with 5% CO_2_ at 37 °C using low-glucose Dulbecco’s Modified Eagle’s Medium (DMEM, Gbico, Waltham, MA, USA) containing 10% fetal bovine serum (Hyclone, Logan, UT, USA), 2 mM glutamine, penicillin (100 U/mL), Solarbio Life Sciences, Beijing, China) and streptomycin (100 μg/mL, Solarbio Life Sciences, Beijing, China). The MSCs showed a long spindle shape and were positive for the surface antigens CD29 and CD54 and negative for the surface antigens CD11b/c and CD45 (Figure S1) that met the standard of ISCT. For all experiments, MSCs from passages 2 to 5 were used.

### 2.3. Mito-TEMPO (MT) and VP Treatment

To eliminate excessive mitochondrial reactive oxygen species (mtROS) or inhibit YAP in BMSCs, 5 μM MT (Selleck, Shanghai, China) or 3 μM VP (Med Chem Express, NJ, USA) was added to the medium during the SMG rotation.

### 2.4. Senescence-Associated-β-Galactosidase (SA-β-gal) Assay

Cellular senescence was identified by measuring SA-β-gal activity using a commercially available kit (Beyotime, Shanghai, China) per the manufacturer’s protocol. Briefly, cells were washed with PBS and suspended in a fixative solution for 10–15 min at room temperature. MSCs were washed twice with PBS, then a staining solution was added, followed by overnight incubation at 37 °C without CO_2_. Subsequently, MSCs were observed and visualized under a light microscope. The SA-β-gal-positive cells and the total number of MSCs were counted using ImageJ (NIH, Wisconsin, MA, USA).

### 2.5. RNA Isolation and Quantitative Real-Time PCR Analysis

Total RNA was extracted using an RNA extraction kit (TIANGEN, Beijing, China) and reverse-transcribed into cDNA using an iScript cDNA synthesis kit (TaKaRa, Tokyo, Japan). The primers used are listed in Table 1. The real-time polymerase chain reaction was performed using SYBR Green PCR mix (Bimake, San Francisco, CA, USA) in a real-time PCR apparatus (Bio-Rad, Hercules, CA, USA). GAPDH was used as an internal control. The accession numbers are as follows: p53 NM_030989.3, p21 U24174.1, p16 L81167.1, and GAPDH NM_017008.4.

### 2.6. Western Blot

Total proteins were extracted with RIPA Lysis (Beyotime, Shanghai, China). Equal amounts of protein were separated by 10% or 12% sodium dodecyl sulfate-polyacrylamide gel electrophoresis (SDS-PAGE) and wet-blotted on polyvinylidene fluoride (PVDF) membranes (Bio-Rad, Hercules, CA, USA). Blocked in 5% non-fat milk (Biosharp, Shanghai, China) for 1 h, the membranes were incubated with primary antibodies (YAP1, 1:1000, Cell Signaling Technology, Danvers, MA, USA; p53, 1:1000, Cell Signaling Technology, Danvers, MA, USA; p21, 1:1000, Abcam, Cambridge, UK; p16, 1:2500, Abcam, Cambridge, UK; GAPDH,1:5000, ZEN-BIOSCIENCE, Chengdu, China). Protein bands were stained using HRP-conjugated secondary antibodies (goat anti-rabbit IgG-HRP, 1:5000, ZBGB-BIO, Beijing, China; goat anti-mouse IgG-HRP, 1:5000, ZBGB-BIO, Beijing, China). Membranes were developed with Plus-ECL (Bio-rad, Hercules, CA, USA) substrates and exposed to a luminescent image analyzer (Bio-OI, Guangzhou, China). Quantitative densitometry of the immunoreactive bands was performed using ImageJ.

### 2.7. Measurement of Intracellular ROS

After respective treatments, intracellular ROS were measured by ROS assay kit (Beyotime, Shanghai, China) according to the manufacturer’s introduction. Briefly, cells were washed 1–2 times with PBS (Gbico, MA, USA) and stained with 10 μM 2′,7′-dichlorofluorescein diacetate (DCFH-DA) for 20 min at 37 °C in a cell incubator. The cells were washed 3 times with serum-free DMEM to remove DCFH-DA that had not entered the cells adequately. Finally, photographs were taken using fluorescence microscopy (CKX53, Olympus), and the results were analyzed using ImageJ.

### 2.8. Measurement of mtROS

After respective treatments, mtROS were evaluated via a fluorescence microscope using red mitochondrial superoxide indicator (MitoSOX) fluorescent dye (Yeasen, Shanghai, China) based on the manufacturer’s instructions. The MSCs were washed 1–2 times with PBS and stained with MitoSOX (5 μM) for 10 min at 37 °C. Then the MSCs were washed 3 times with pre-warmed PBS. Finally, photographs were taken using fluorescence microscopy, and the experimental results were analyzed using ImageJ.

### 2.9. Mitochondrial Membrane Potential (mΔΨm) Assay

After respective treatments, the mΔΨm was evaluated by using MitoTracker Red fluorescent dye (Invitrogen, Carlsbad, CA, USA) and MitoTracker Green (Yeasen, Shanghai, China) based on the manufacturer’s instructions. The higher the mΔΨm is, the more MitoTracker Red aggregates that form, which appear with a red fluorescence, in contrast to the MitoTracker Green, which has a green fluorescence. Thus, the mΔΨm was displayed by the change in the ratio between red and green fluorescence. The MSCs were collected and stained with MitoTracker Red (100 nM) and MitoTracker Green (200 nM) for 30 min at 37 °C in the dark and then determined using fluorescence microscopy. The results were analyzed using ImageJ.

### 2.10. Measurement of ATP

MSCs were lysed using the lysis solution provided in the ATP assay kit (Beyotime, Shanghai, China), centrifuged at 12,000 rpm for 5 min at 4 °C, and the supernatant was collected. The samples’ chemiluminescence intensity and protein concentration were measured by a microplate luminometer (BioTek, Thorold, ON, Canada). The ATP content was normalized to protein concentration, and the results were expressed as fold change compared with the control group.

### 2.11. Immunofluorescence (IF) Staining

IF staining was used to detect the distribution and expression of YAP in MSCs. Briefly, the 4% paraformaldehyde-fixed cells were blocked with 1% BSA. After that, the sections were probed with the primary antibody against YAP1 rabbit monoclonal antibody (1:100, Cell Signaling Technology, Danvers, MA, USA) at 4 °C overnight. After incubation with secondary antibodies conjugated to Fluor-488 (1:200, ZEN-BIOSCIENCE, Chengdu, China) for 1 h at room temperature. Nuclei were visualized by staining with DAPI (Biosharp, Shanghai, China) for 5 min at room temperature. Finally, photographs were taken using fluorescence microscopy, and the results were analyzed using ImageJ.

### 2.12. Statistical Analysis

Prism v8.0 software was used to prepare graphs and statistics. All the values were presented as mean ± SEM, and for each assay, a minimum of three independent experimental repeats were performed. Student’s t-test was used to analyze the differences between the two groups, and one-way ANOVA was used for multiple comparations. *p* < 0.05 was considered significant.

## 3. Results

### 3.1. Construction of SMG

A 2D-clinostat device was used to simulate microgravity. The fixed chamber was constructed utilizing a cell culture slide, a silicon seal, a gas-permeable polystyrene membrane, and a polycarbonate base (Figure 1A). In the clinostat device, the orientation of the MSC changed constantly, and the gravity vector changed accordingly. The principle of using a clinostat to model simulated microgravity is illuminated as follows: Suppose cells adhered to the slide; when the sample rotates by 90° (from position 1 to position 2), the gravitational vector will change from (0, −) to (+, 0) (Figure 1B). In a complete 360° process, the average gravitational vector cell sense reduces to approximately 0 g. Under these conditions, cells cannot feel gravity, and the gravity vector escapes its detection machinery. In this study, the device produced a simulated gravity of about 10^−3^ g by rotating at 10 rpm.

### 3.2. SMG Induced MSC Senescence

**Figure 2:**
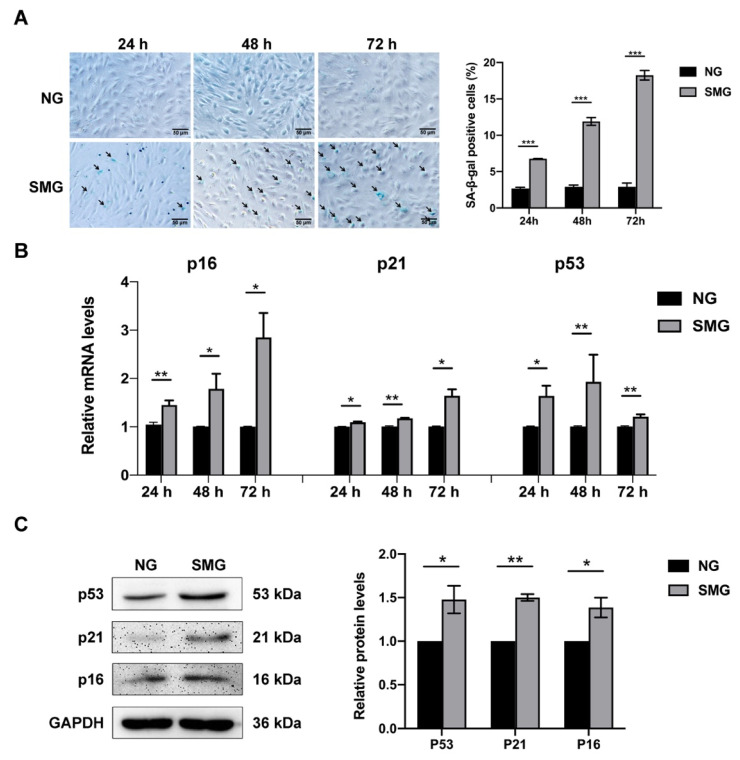
SMG induced the senescence of MSCs. (**A**) The representative images and quantitative analysis of SA-β-gal staining after 24 h, 48 h, and 72 h of treatment under NG or SMG (bar = 50 μm). (**B**) The mRNA expression of p53, p21, and p16 in MSCs after 24 h, 48 h, and 72 h of treatment under NG or SMG (**C**) The representative images and quantitative analysis of the protein level expression of p53, p21, and p16 in MSCs after 72 h of treatment under NG or SMG. NG—normal gravity; SMG—simulated microgravity; *n* = 3, * *p* < 0.05, ** *p* < 0.01, *** *p* < 0.001.

To investigate whether SMG induces the senescence of MSCs, MSCs were exposed to SMG for 24 h, 48 h, and 72 h, and the SA-β-gal activity and the expression of senescence markers were detected. The SA-β-gal activity was expressed as the percentage of SA-β-gal-positive cells. The activity of SA-β-gal increased at 24 h and gradually upregulated with the prolongation of SMG treatment time (Figure 2A). Meanwhile, we detected a marked increase in the mRNA expression of senescence markers in SMG-induced MSCs, including the cyclin-dependent kinase inhibitors p21 and p16 and the cell cycle arrest protein p53 (Figure 2B). The protein level expression of senescent markers was confirmed by Western blot at 72 h of SMG treatment and demonstrated that the protein expression of senescence markers in SMG-induced MSCs was significantly increased (Figure 2C). Thus, the following studies were performed using MSCs subjected to SMG for 72 h. Our results showed that SMG induced MSC senescence.

### 3.3. SMG-Induced Mitochondrial Dysfunction in MSCs

**Figure 3:**
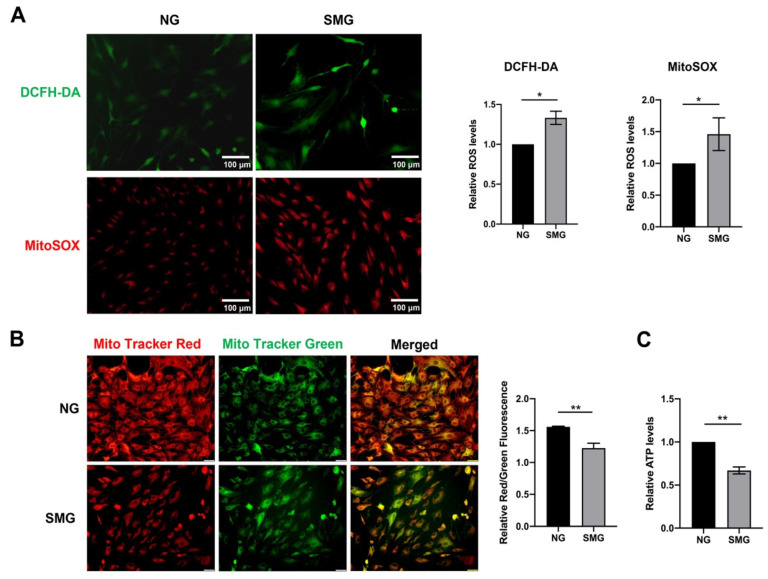
SMG-induced mitochondrial dysfunction in MSCs. (**A**) The representative images and quantitative analysis of the intracellular ROS and mtROS, which were determined by DCFH-DA and MitoSOX staining in MSCs. (bar = 100 μm). (**B**) The representative images and quantitative analysis of the mΔΨm detected by MitoTracker Red and MitoTracker Green staining in MSCs. The mΔΨm was displayed by the change in the ratio between red and green fluorescence (bar = 100 μm). (**C**) The bar graph displayed the ATP levels in MSCs. NG—normal gravity; SMG—simulated microgravity; *n* = 3, * *p* < 0.05, ** *p* < 0.01.

To determine the effect of SMG on mitochondrial function, the mtROS, intercellular ROS, mΔΨm, and ATP levels were investigated. MitoSOX and DCFH-DA staining demonstrated that increased mtROS and intercellular ROS levels were in MSCs under SMG for 72 h (Figure 3A). The mΔΨm of MSCs were notably reduced, which was detected using MitoTracker Red and MitoTracker Green staining (Figure 3B). At the same time, reduced ATP production was observed in MSCs under SMG. These findings suggested SMG-induced mitochondrial dysfunction in MSCs. Remarkably, mtROS level was increased about twofold in MSCs, suggesting a link between oxidative stress and cellular senescence in MSCs (Figure 3A).

### 3.4. MT Attenuated SMG-Induced Mitochondrial Dysfunction by Reducing mtROS in MSCs

**Figure 4:**
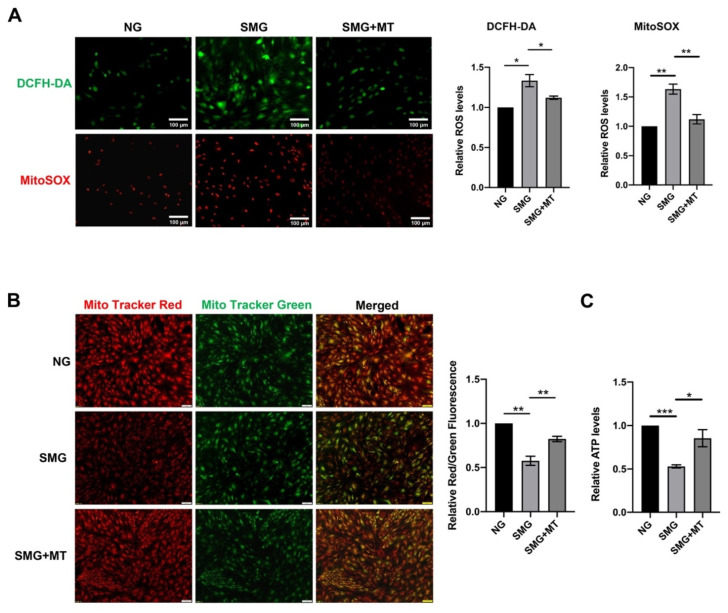
MT attenuated mitochondrial dysfunction in MSCs under SMG. (**A**) The representative images and quantitative analysis of the intracellular ROS and mtROS in MSCs which were determined by DCFH-DA and MitoSOX staining (bar = 100 μm). (**B**) The representative images and quantitative analysis of the mΔΨm in MSCs were detected by MitoTracker Red and MitoTracker Green staining (bar = 100 μm). (**C**) The bar graph displayed the ATP levels in MSCs. NG—normal gravity; SMG—simulated microgravity; MT—Mito-TEMPO; *n* = 3, * *p* < 0.05, ** *p* < 0.01, *** *p* < 0.001.

Mitochondria are the primary source of ROS production [35]. To further explore the relationship between mitochondrial dysfunction and SMG-induced MSC senescence, we eliminated the excess mtROS by MT, a mitochondrial antioxidant. MitoSOX and DCFH-DA staining demonstrated that MT treatment significantly attenuated the increased mtROS and intercellular ROS levels in MSCs under SMG and reversed SMG-induced decrease in mΔΨm and ATP levels in MSCs (Figure 4A–C). These findings suggest that MT restores mitochondrial dysfunction affected by SMG via eliminating mtROS in MSCs.

### 3.5. MT Attenuated SMG-Induced MSC Senescence

**Figure 5:**
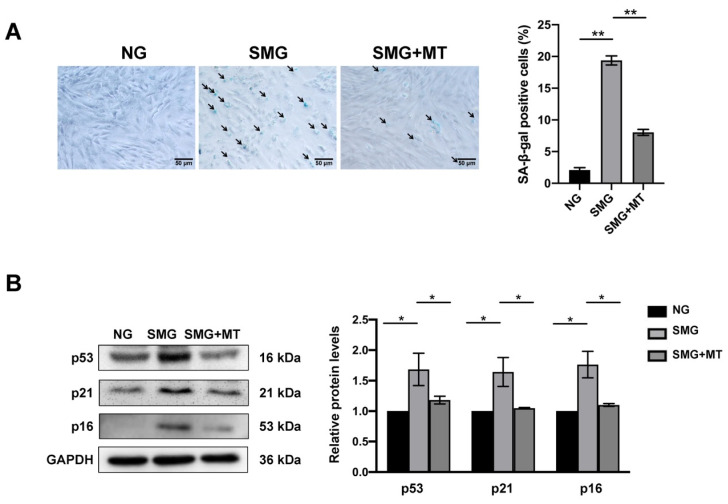
MT attenuated the senescence of MSCs under SMG. (**A**) The representative images and quantitative analysis of SA-β-gal staining in MSCs (bar = 50 μm). (**B**) The representative images and quantitative analysis of the protein level expression of p53, p21, and p16 in MSCs. NG—normal gravity; SMG—simulated microgravity; MT—Mito-TEMPO; *n* = 3, * *p* < 0.05, ** *p* < 0.01.

ROS-induced oxidative stress is an important cause of cell senescence [36]. We next examined the effect of MT on the senescence of MSCs. We found that MSC senescence under SMG could be readily reduced after MT treatment, which was evidenced by not only a decrease in SA-β-gal staining but also a reduction in the expression of cellular senescence markers, including p53, p21, and p16 (Figure 5A,B). In addition, we used β-actin as a secondary loading control to confirm whether GAPDH stably expressed after treatment and found that the expressions of GAPDH and β-actin were unchanged by treatment (Figure S2). In aggregate, our results demonstrated MT attenuates SMG-induced MSC senescence and suggested that excessive ROS production from mitochondrial dysfunction mediates this process.

### 3.6. VP Attenuated Mitochondrial Dysfunction by Inhibiting YAP in MSCs

**Figure 6:**
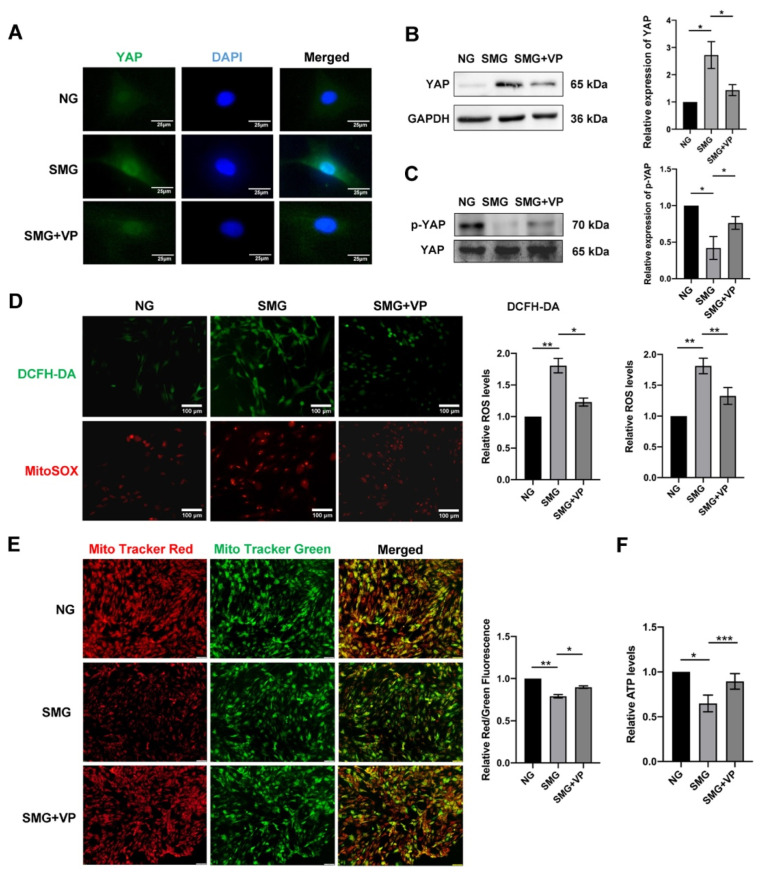
YAP inhibition by VP reduced mitochondrial dysfunction in MSCs under SMG. (**A**) The representative images of IF staining of YAP (green) and DAPI (blue) in MSCs (bar = 25 μm). (**B**) The representative images and quantitative analysis of the protein level expression of YAP in MSCs. (**C**) The representative images and quantitative analysis of the protein level expression of p-YAP in MSCs. (**D**) The representative images and quantitative analysis of the intracellular ROS and mtROS in MSCs which were determined by DCFH-DA and MitoSOX staining (bar = 100 μm). (**E**) The representative images and quantitative analysis of the mΔΨm in MSCs were detected by MitoTracker Red and MitoTracker Green staining (bar = 100 μm). (**F**) The bar graph displayed the ATP levels in MSCs. NG—normal gravity; SMG—simulated microgravity; VP—verteporfin; *n* = 3, * *p* < 0.05, ** *p* < 0.01, *** *p* < 0.001.

To determine whether YAP is associated with SMG-induced MSC senescence, IF and Western blot were used to detect the expression and nuclear localization of YAP. The result showed that the expression and nuclear localization of YAP were significantly increased in SMG-induced MSCs. To further explore the relationship between YAP and mitochondrial dysfunction, VP, a YAP inhibitor, was used to inhibit YAP expression and nuclear translocation (Figure 6A–C). The mitochondrial function of MSCs after the inhibitor VP treatment under SMG was examined. We found that VP treatment significantly reduced mtROS and intercellular ROS levels but upregulated the mΔΨm in MSCs exposed to SMG for 72 h (Figure 6D,E). VP treatment also markedly enhanced the level of ATP in SMG-induced MSCs (Figure 6F). These results suggested that YAP mediates SMG-induced mitochondrial dysfunction in MSCs.

### 3.7. VP Attenuated SMG-Induced MSC Senescence

**Figure 7:**
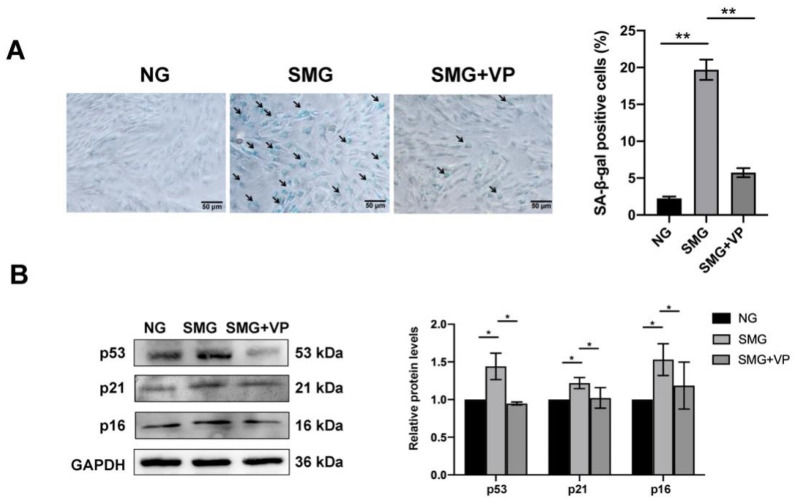
YAP inhibition by VP alleviated the senescence of MSCs under SMG. (**A**) The representative images and quantitative analysis of SA-β-gal staining in MSCs (bar = 50 μm). (**B**) The representative images and quantitative analysis of the protein level expression of p53, p21, and p16 in MSCs. NG—normal gravity; SMG—simulated microgravity; VP—verteporfin; *n* = 3, * *p* < 0.05, ** *p* < 0.01.

To test whether YAP mediates SMG-induced MSC senescence by regulating mitochondrial dysfunction, we investigated SA-β-gal activity in MSCs and found a decrease in the percentage of SA-β-gal-positive cells in VP-treated MSCs under SMG (Figure 7A). Coherently, VP inhibition of YAP also rescued the expression of cellular senescence markers, p53, p21, and p16 in MSCs (Figure 7B). Together with the finding that VP attenuates SMG-induced mitochondrial dysfunction, these observations demonstrated that YAP upregulation mediated SMG-induced MSC senescence by regulating mitochondrial dysfunction.

## 4. Discussion

**Figure 8:**
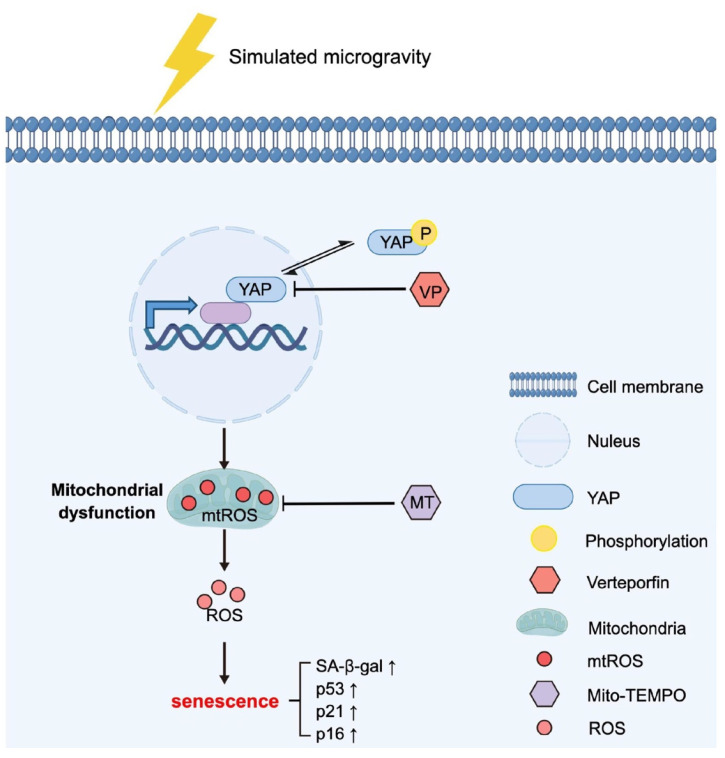
The mechanism of YAP-mediated promoting MSC senescence under SMG. Upon mechanical stimulation of SMG, YAP expression and nuclear localization were significantly elevated, which led to excess mtROS and intracellular ROS via inducing mitochondrial dysfunction and further contributed to MSC senescence. The addition of YAP inhibitor VP or mitochondrial antioxidant MT effectively alleviates mitochondrial dysfunction and MSC senescence.

MSCs are a kind of adult stem cells with multi-directional differentiation ability, which contribute to repairing damaged or diseased tissues and organs. However, little is known about the effects of microgravity on MSC senescence and related mechanisms. In this study, using a 2D-clinostat device to simulate microgravity, we revealed the crucial role of YAP in SMG-induced MSC senescence (Figure 8). We found that mitochondrial dysfunction mediates SMG-induced MSC senescence, and MT restores mitochondrial dysfunction affected by SMG via eliminating mtROS in MSCs. Further, we found SMG promoted YAP expression and its nuclear translocation in MSCs, and VP, an inhibitor of YAP, restored SMG-induced mitochondrial dysfunction and senescence in MSCs by inhibiting YAP expression and nuclear localization. Our results also suggest that YAP is a possible target for the treatment of weightlessness-related cell senescence and aging, and in addition to antioxidants MT, the YAP inhibitor VP also plays a good anti-senescence role.

MSC-based treatments have shown good therapeutic potential in a variety of diseases in preclinical studies and clinical trials [37,38]. However, even under NG conditions, MSCs are prone to senescence with the increase in in vitro passages, thereby reducing their beneficial effects, not to mention the complex space microgravity environment [39,40]. The senescent MSCs exhibit growth arrest, enlarged cell size, increased lysosomal content characterized by SA-β-gal, and lower proliferative capacity [41]. The activity of p53, inducing a downstream gene p21, is positively correlated with cellular senescence [42]. P16 is a cell cycle inhibitor protein, one of the important pathways to activate DDR, and plays an important role in premature aging and cellular senescence [43]. In this study, we showed that MSCs developed into the senescent phenotype during SMG induction, as evidenced by enhanced SA-β-gal positivity and elevation of the p53, p21, and p16 levels.

The cause or consequence of mitochondrial dysfunction is increased mtROS levels and oxidative stress products, which are one of the main drivers of the cell senescence process [44,45]. It has been proved that salivary gland epithelial cell senescence and mitochondrial dysfunction could be inhibited by mitochondria-targeted mtROS scavenger MT which eliminates accumulated mtROS [46]. Another study also found that the supplementation of nicotinamide adenine dinucleotide (NAD^+^) can improve mitochondrial function and prolong the life span of mice [47]. In addition, some scholars have found that mitochondrial dysfunction resulted in ROS in MSCs, and ROS inhibition via N-acetylcysteine (NAC), a widely used inhibitor of ROS, successfully rescued MSCs from senescence [48]. Consistent with published studies, mitochondrial antioxidant MT effectively inhibited SMG-induced MSC mitochondrial dysfunction and senescence by suppressing the accumulation of mtROS in this study.

YAP is a core effector of the Hippo signaling pathway, and its expression and nuclear/cytoplasmic distribution are regulated by mechanical factors such as matrix stiffness and shear stress [49]. Some studies have proved that the activity of YAP is affected by microgravity. Arun et al. showed that YAP expression decreased, but nuclear localization increased following SMG treatment of polyploid giant cancer cells [50]. Moreover, Camberos et al. showed that spaceflight and SMG increased the YAP expression in cardiovascular progenitors [51]. Consistent with Camberos et al., our work suggests that SMG upregulates the YAP expression and leads to MSC mitochondrial dysfunction and senescence. An increasing number of studies have shown that YAP is a critical regulator in controlling longevity [22,23,24,25,26,27,28,29,30]. Compared with young rats, aging rats have higher expression of YAP in vascular tissue [26]. The activation of the Hippo signaling pathway induces mitochondrial dysfunction in mice, and the reduction in YAP nuclear translocalization inhibits the transcription of a large number of mitochondrial genes involved in mitochondrial turnover and metabolism [52]. Increased nuclear translocalization of YAP can also lead to mitochondrial dysfunction and further inhibits cell differentiation and growth or even promotes apoptosis [53,54,55]. On the other hand, it has been reported that mitochondrial dysfunction also decreases the nuclear translocalization of YAP and disrupts the self-renewal and lineage differentiation of human pluripotent stem cells [56]. Our results suggest that YAP is highly likely to mediate SMG-induced MSC senescence by promoting mitochondrial dysfunction. In addition, a study has shown that VP, a YAP inhibitor, inhibits YAP expression by interfering with YAP/TEAD and alleviates the aging of YAP transgenic mice [57]. Another study also found that VP treatment remittances the senescence of endothelial cells and vascular tissue by inhibiting YAP expression [27]. Indeed, in the current study, we found that the inhibition of YAP using VP attenuated mitochondrial dysfunction and suppressed SMG-induced senescence in MSCs. Our results demonstrate that YAP inhibition alleviates SMG-induced MSC senescence via targeting mitochondrial dysfunction, suggesting an idea of therapeutic treatment of cell senescence under microgravity by targeting YAP.

In addition to YAP, a number of mechanotransducers were studied under a gravity-unloading environment, including F-actin, NK-κB, and β-catenin [58,59,60]. In most of the studies, the responses of molecules in signaling transduction are similar under SMG and real microgravity in space. However, some exceptions are also found. For example, F-actin, an extensively studied mechano-transducer, was found to have an increased polymerization in cells experiencing spaceflight or SMG, which is contrary to most studies [61,62,63]. Likewise, tubulin in the arterial endothelial cells (EA. hy 926 cell line) performs completely differently under real microgravity or SMG [64,65]. These results indicate that it is crucial for an extensive comparison of mechanotransduction under SMG and real microgravity in further study.

## 5. Conclusions

In conclusion, we demonstrated that SMG induced the senescence of MSCs by upregulating YAP expression and nuclear translocation to promote mitochondrial dysfunction. The addition of YAP inhibitor VP or mitochondrial antioxidant MT effectively alleviates mitochondrial dysfunction and MSC senescence. Our results propose the role of YAP in SMG-induced MSC senescence and indicate that YAP may be a potential therapeutic target for treating space aging-related diseases.

## Figures and Tables

**Table 1:** Primers sequence for real-time PCR.

Genes	Forward Primer Sequence (5′-3′)	Forward Primer Sequence (3′-5′)
P53	CCAGGATGTTGCAGAGTTGTTAGA	TTGAGAAGGGACGGAAGATGAC
P21	GGGACAGCAGAGGAAGACC	GACTAAGGCAGAAGATGTAGAGC
P16	CTCCTTGGCTTCATTCTGG	TCCAATCGTCTCCCTCCCTC
GAPDH	TGACTTCAACAGCGACACCCA	CACCCTGTTGCTGTAGCCAAA

## Data Availability

Data are contained within the article and Supplementary Materials.

## Supplementary Materials

The following supporting information can be downloaded at: https://www.mdpi.com/article/10.3390/antiox12050990/s1. Figure S1: Identification of MSCs. (A) The P0-P3 MSCs showed a long spindle shape (bar = 100 μm). (B) MSCs surface antigen identification by flow cytometry. MSCs: mesenchymal stem cells; Negative antigen: CD11b/c and CD45; positive antigen: CD29 and CD54; Figure S2: The representative image (A) and the quantitative analysis of the protein level expression of β-actin by normalizing to GADPH (B). MSCs: mesenchymal stem cells; NG: normal gravity; SMG: simulated microgravity; MT: Mito-TEMPO; *n* = 3, ns *p* > 0.05.

## References

[1] Tharp K.M., Higuchi-Sanabria R., Timblin G.A., Ford B., Garzon-Coral C., Schneider C., Muncie J.M., Stashko C., Daniele J.R., Moore A.S. (2021). Adhesion-mediated mechanosignaling forces mitohormesis. Cell Metab..

[2] Duan J.L., Ruan B., Song P., Fang Z.Q., Yue Z.S., Liu J.J., Dou G.R., Han H., Wang L. (2022). Shear stress-induced cellular senescence blunts liver regeneration through Notch-sirtuin 1-P21/P16 axis. Hepatology.

[3] Konstantonis D., Papadopoulou A., Makou M., Eliades T., Basdra E., Kletsas D. (2014). The role of cellular senescence on the cyclic stretching-mediated activation of MAPK and ALP expression and activity in human periodontal ligament fibroblasts. Exp. Gerontol..

[4] Kandarpa K., Schneider V., Ganapathy K. (2019). Human health during space travel: An overview. Neurol. India.

[5] Prasad B., Grimm D., Strauch S.M., Erzinger G.S., Corydon T.J., Lebert M., Magnusson N.E., Infanger M., Richter P., Krüger M. (2020). Influence of microgravity on apoptosis in cells, tissues, and other systems in vivo and in vitro. Int. J. Mol. Sci..

[6] Dinarelli S., Longo G., Dietler G., Francioso A., Mosca L., Pannitteri G., Boumis G., Bellelli A., Girasole M. (2018). Erythrocyte’s aging in microgravity highlights how environmental stimuli shape metabolism and morphology. Sci. Rep..

[7] Takahashi H., Nakamura A., Shimizu T. (2021). Simulated microgravity accelerates aging of human skeletal muscle myoblasts at the single cell level. Biochem. Biophys. Res. Commun..

[8] Carlsson S.I., Bertilaccio M.T., Ballabio E., Maier J.A. (2003). Endothelial stress by gravitational unloading: Effects on cell growth and cytoskeletal organization. Biochim. Biophys. Acta.

[9] Wang J., Zhang J., Bai S., Wang G., Mu L., Sun B., Wang D., Kong Q., Liu Y., Yao X. (2009). Simulated microgravity promotes cellular senescence via oxidant stress in rat pc12 cells. Neurochem. Int..

[10] Lan T., Luo M., Wei X. (2021). Mesenchymal stem/stromal cells in cancer therapy. J. Hematol. Oncol..

[11] Harrell C.R., Jovicic N., Djonov V., Arsenijevic N., Volarevic V. (2019). Mesenchymal stem cell-derived exosomes and other extracellular vesicles as new remedies in the therapy of inflammatory diseases. Cells.

[12] Turinetto V., Vitale E., Giachino C. (2016). Senescence in human mesenchymal stem cells: Functional changes and implications in stem cell-based therapy. Int. J. Mol. Sci..

[13] Demontis G.C., Germani M.M., Caiani E.G., Barravecchia I., Passino C., Angeloni D. (2017). Human pathophysiological adaptations to the space environment. Front. Physiol..

[14] Tran K.N., Choi J.I. (2022). Mimic Microgravity Effect on muscle transcriptome under ionizing radiation. Life Sci. Space Res..

[15] Patel S. (2020). The effects of microgravity and space radiation on cardiovascular health: From low-earth orbit and beyond. Int. J. Cardiol. Heart Vasc..

[16] Loi F., Córdova L.A., Pajarinen J., Lin T.H., Yao Z., Goodman S.B. (2016). Inflammation, fracture and bone repair. Bone.

[17] Pajarinen J., Lin T., Gibon E., Kohno Y., Maruyama M., Nathan K., Lu L., Yao Z., Goodman S.B. (2019). Mesenchymal stem cell-macrophage crosstalk and bone healing. Biomaterials.

[18] Kimoloi S., Sen A., Guenther S., Braun T., Brügmann T., Sasse P., Wiesner R.J., Pla-Martín D., Baris O.R. (2022). Combined fibre atrophy and decreased muscle regeneration capacity driven by mitochondrial DNA alterations underlie the development of sarcopenia. J. Cachexia Sarcopenia Muscle.

[19] Wu L.W., Wang Y.L., Christensen J.M., Khalifian S., Schneeberger S., Raimondi G., Cooney D.S., Lee W.P., Brandacher G. (2014). Donor age negatively affects the immunoregulatory properties of both adipose and bone marrow derived mesenchymal stem cells. Transpl. Immunol..

[20] Moya I.M., Halder G. (2019). Hippo-YAP/TAZ signaling in organ regeneration and regenerative medicine. Nat. Rev. Mol. Cell Biol..

[21] Pobbati A.V., Hong W. (2020). A Combat with the YAP/TAZ-TEAD oncoproteins for cancer therapy. Theranostics.

[22] Piccolo S., Dupont S., Cordenonsi M. (2014). The biology of YAP/TAZ: Hippo signaling and beyond. Physiol. Rev..

[23] Xu J., Tang Y., Sheng X., Tian Y., Deng M., Du S., Lv C., Li G., Pan Y., Song Y. (2020). Secreted stromal protein ISLR promotes intestinal regeneration by suppressing epithelial hippo signaling. EMBO J..

[24] Childs B.G., Gluscevic M., Baker D.J., Laberge R.M., Marquess D., Dananberg J., Deursen J.M. (2017). Senescent cells: An emerging target for diseases of ageing. Nat. Rev. Drug Discov..

[25] Xie Q., Chen J., Feng H., Peng S., Adams U., Bai Y., Huang L., Li J., Huang J., Meng S. (2013). YAP/TEAD-mediated transcription controls cellular senescence. Cancer Res..

[26] Liu J., Huang K., Cai G.-Y., Chen X.-M., Yang J.-R., Lin L.-R., Yang J., Huo B.-G., Zhan J., He Y.-N. (2014). Receptor for advanced glycation end-products promotes premature senescence of proximal tubular epithelial cells via activation of endoplasmic reticulum stress-dependent p21 signaling. Cell. Signal..

[27] Yeung Y.T., Guerrero-Castilla A., Cano M., Muñoz M.F., Ayala A., Argüelles S. (2019). Dysregulation of the hippo pathway signaling in aging and cancer. Pharmacol. Res..

[28] Fausti F., Di A.S., Cioce M., Bielli P., Sette C., Pandolfi P.P., Oren M., Sudol M., Strano S., Blandino G. (2013). ATM Kinase Enables the functional axis of YAP, PML and p53 to ameliorate loss of werner protein-mediated oncogenic senescence. Cell Death Differ..

[29] Pan X., Wu B., Fan X., Xu G., Ou C., Chen M. (2021). YAP accelerates vascular senescence via blocking autophagic flux and activating mTOR. J. Cell. Mol. Med..

[30] Gong Y., Li S.J., Liu R., Zhan J.F., Tan C., Fang Y.F., Chen Y., Yu B. (2019). Inhibition of YAP with SiRNA prevents cartilage degradation and ameliorates osteoarthritis development. J. Mol. Med..

[31] Xu X., Shen X., Wang J., Feng W., Wang M., Miao X., Wu Q., Wu L., Wang X., Ma Y. (2021). YAP prevents premature senescence of astrocytes and cognitive decline of alzheimer’s disease through regulating CDK6 signaling. Aging Cell.

[32] Xu X., Shen X., Feng W., Yang D., Jin L., Wang J., Wang M., Ting Z., Xue F., Zhang J. (2020). D-galactose induces senescence of glioblastoma cells through YAP-CDK6 pathway. Aging.

[33] Jin H., Lian N., Bian M., Zhang C., Chen X., Shao J., Wu L., Chen A., Guo Q., Zhang F. (2018). Oroxylin A inhibits ethanol-induced hepatocyte senescence via YAP pathway. Cell Prolif..

[34] Dupont S., Morsut L., Aragona M., Enzo E., Giulitti S., Cordenonsi M., Zanconato F., Le D.J., Forcato M., Bicciato S. (2011). Role of YAP/TAZ in mechanotransduction. Nature.

[35] Zhou L., Li R., Liu C., Sun T., Htet A.L.H., Chen C., Gao J., Zhao Y., Wang K. (2017). Foxo3a inhibits mitochondrial fission and protects against doxorubicin-induced cardiotoxicity by suppressing MIEF2. Free Radic. Biol. Med..

[36] Davalli P., Mitic T., Caporali A., Lauriola A., D’Arca D. (2016). ROS, cell senescence, and novel molecular mechanisms in aging and age-related diseases. Oxidative Med. Cell. Longev..

[37] Zhang Y., Yu Z., Jiang D., Liang X., Liao S., Zhang Z., Yue W., Li X., Chiu S.M., Chai Y.H. (2016). Ipsc-mscs with high intrinsic MIRO1 and sensitivity to TNF-α yield efficacious mitochondrial transfer to rescue anthracycline-induced cardiomyopathy. Stem Cell Rep..

[38] Liang X., Ding Y., Zhang Y., Chai Y.H., He J., Chiu S.M., Gao F., Tse H.F., Lian Q. (2015). Activation of NRG1-ERBB4 signaling potentiates mesenchymal stem cell-mediated myocardial repairs following myocardial infarction. Cell Death Dis..

[39] Zhang T., Wang P., Liu Y., Zhou J., Shi Z., Cheng K., Huang T., Wang X., Yang G.L., Yang B. (2018). Overexpression of FOXQ1 enhances anti-senescence and migration effects of human umbilical cord mesenchymal stem cells in vitro and in vivo. Cell Tissue Res..

[40] Kim J., Kim Y., Choi H., Kwon A., Jekarl D.W., Lee S., Jang W., Chae H., Kim J.R., Kim J.M. (2018). Ubiquitin C decrement plays a pivotal role in replicative senescence of bone marrow mesenchymal stromal cells. Cell Death Dis..

[41] Lunyak V.V., Amaro-Ortiz A., Gaur M. (2017). Mesenchymal stem cells secretory responses: Senescence messaging secretome and immunomodulation perspective. Front. Genet..

[42] Jiang C., Liu G., Luckhardt T., Antony V., Zhou Y., Carter A.B., Thannickal V.J., Liu R.M. (2017). Serpine 1 induces alveolar type II cell senescence through activating p53-p21-Rb pathway in fibrotic lung disease. Aging Cell.

[43] Maggiorani D., Manzella N., Edmondson D.E., Mattevi A., Parini A., Binda C., Mialet-Perez J. (2017). Monoamine oxidases, oxidative stress, and altered mitochondrial dynamics in cardiac ageing. Oxidative Med. Cell. Longev..

[44] Weng Z., Wang Y., Ouchi T., Liu H., Qiao X., Wu C., Zhao Z., Li L., Li B. (2022). Mesenchymal stem/stromal cell senescence: Hallmarks, mechanisms, and combating strategies. Stem Cells Transl. Med..

[45] Kudryavtseva A.V., Krasnov G.S., Dmitriev A.A., Alekseev B.Y., Kardymon O.L., Sadritdinova A.F., Fedorova M.S., Pokrovsky A.V., Melnikova N.V., Kaprin A.D. (2016). Mitochondrial dysfunction and oxidative stress in aging and cancer. Oncotarget.

[46] Zhu M., Min S., Mao X., Zhou Y., Zhang Y., Li W., Li L., Wu L., Cong X., Yu G. (2022). Interleukin-13 promotes cellular senescence through inducing mitochondrial dysfunction in igg4-related sialadenitis. Int. J. Oral. Sci..

[47] Aebersold R., Schoonjans K., Menzies K.J., Auwerx J. (2016). NAD^+^ repletion improves mitochondrial and stem cell function and enhances life span in mice. Science.

[48] Ye G., Xie Z., Zeng H., Wang P., Li J., Zheng G., Wang S., Cao Q., Li M., Liu W. (2020). Oxidative stress-mediated mitochondrial dysfunction facilitates mesenchymal stem cell senescence in ankylosing spondylitis. Cell Death Dis..

[49] Seo J., Kim J. (2018). Regulation of hippo signaling by actin remodeling. BMB Rep..

[50] Arun R.P., Sivanesan D., Patra B., Varadaraj S., Verma R.S. (2019). Simulated microgravity increases polyploid giant cancer cells and nuclear localization of YAP. Sci. Rep..

[51] Camberos V., Baio J., Bailey L., Hasaniya N., Lopez L.V., Kearns-Jonker M. (2019). Effects of spaceflight and simulated microgravity on YAP1 expression in cardiovascular progenitors: Implications for cell-based repair. Int. J. Mol. Sci..

[52] Wu W., Ziemann M., Huynh K., She G., Pang Z.D., Zhang Y., Duong T., Kiriazis H., Pu T.T., Bai R.Y. (2021). Activation of hippo signaling pathway mediates mitochondria dysfunction and dilated cardiomyopathy in mice. Theranostics.

[53] Yu H., Yang Z., Xu M., Huang J., Yue Z., Guo B. (2022). Yap is essential for uterine decidualization through Rrm2/GSH/ROS pathway in response to Bmp2. Int. J. Biol. Sci..

[54] Wang L., Wang C., Tao Z., Zhao L., Zhu Z., Wu W., He Y., Chen H., Zheng B., Huang X. (2019). Curcumin derivative WZ35 inhibits tumor cell growth via ROS-YAP-JNK signaling pathway in breast cancer. J. Exp. Clin. Cancer Res..

[55] Li H., Wang X., Mu H., Mei Q., Liu Y., Min Z., Zhang L., Su P., Xiang W. (2022). Mir-484 contributes to diminished ovarian reserve by regulating granulosa cell function via YAP1-mediated mitochondrial function and apoptosis. Int. J. Biol. Sci..

[56] Qi Y., Ye Y., Wang R., Yu S., Zhang Y., Lv J., Jin W., Xia S., Jiang W., Li Y. (2022). Mitochondrial dysfunction by TFAM depletion disrupts self-renewal and lineage differentiation of human PSCs by affecting cell proliferation and YAP response. Redox Biol..

[57] Marquard S., Thomann S., Weiler S.M.E., Bissinger M., Lutz T., Sticht C., Tóth M., De L.T.C., Gretz N., Straub B.K. (2020). Yes-associated protein (YAP) induces a secretome phenotype and transcriptionally regulates plasminogen activator inhibitor-1 (PAI-1) expression in hepatocarcinogenesis. Cell Commun. Signal..

[58] Qian A.R., Li D., Han J., Gao X., Di S.M., Zhang W., Hu L.F., Shang P. (2012). Fractal dimension as a measure of altered actin cytoskeleton in MC3T3-E1 cells under simulated microgravity using 3-D/2-D clinostats. IEEE Trans. Biomed. Eng..

[59] Zhao T., Li R., Tan X., Zhang J., Fan C., Zhao Q., Deng Y., Xu A., Lukong K.E., Genth H. (2018). Simulated Microgravity Reduces Focal Adhesions and Alters Cytoskeleton and Nuclear Positioning Leading to Enhanced Apoptosis via Suppressing FAK/RhoA-Mediated mTORC1/NF-κB and ERK1/2 Pathways. Int. J. Mol. Sci..

[60] Fan C., Wu Z., Cooper D.M.L., Magnus A., Harrison K., Eames B.F., Chibbar R., Groot G., Huang J., Genth H. (2022). Activation of Focal Adhesion Kinase Restores Simulated Microgravity-Induced Inhibition of Osteoblast Differentiation via Wnt/B-Catenin Pathway. Int. J. Mol. Sci..

[61] Lü D., Sun S., Zhang F., Luo C., Zheng L., Wu Y., Li N., Zhang C., Wang C., Chen Q. (2019). Microgravity-induced hepatogenic differentiation of rBMSCs on board the SJ-10 satellite. FASEB J..

[62] Nassef M.Z., Kopp S., Wehland M., Melnik D., Sahana J., Krüger M., Corydon T.J., Oltmann H., Schmitz B., Schütte A. (2019). Real Microgravity Influences the Cytoskeleton and Focal Adhesions in Human Breast Cancer Cells. Int. J. Mol. Sci..

[63] Hybel T.E., Dietrichs D., Sahana J., Corydon T.J., Nassef M.Z., Wehland M., Krüger M., Magnusson N.E., Bauer J., Utpatel K. (2020). Simulated Microgravity Influences VEGF, MAPK, and PAM Signaling in Prostate Cancer Cells. Int. J. Mol. Sci..

[64] Grosse J., Wehland M., Pietsch J., Ma X., Ulbrich C., Schulz H., Saar K., Hübner N., Hauslage J., Hemmersbach R. (2012). Short-term weightlessness produced by parabolic flight maneuvers altered gene expression patterns in human endothelial cells. FASEB J..

[65] Grimm D., Infanger M., Westphal K., Ulbrich C., Pietsch J., Kossmehl P., Vadrucci S., Baatout S., Flick B., Paul M. (2009). A delayed type of three-dimensional growth of human endothelial cells under simulated weightlessness. Tissue Eng. Part A.

