# Supplementary material for: YAP Inhibition Alleviates Simulated Microgravity-Induced Mesenchymal Stem Cell Senescence via Targeting Mitochondrial Dysfunction
Source: Antioxidants (Basel). 2023 Apr 24;12(5):990. doi: 10.3390/antiox12050990 (PMC10215363; doi:10.3390/antiox12050990)
Supplement: Supplementary file 1 [file antioxidants-12-00990-s001.zip › antioxidants-2316502-supplementary.pdf]

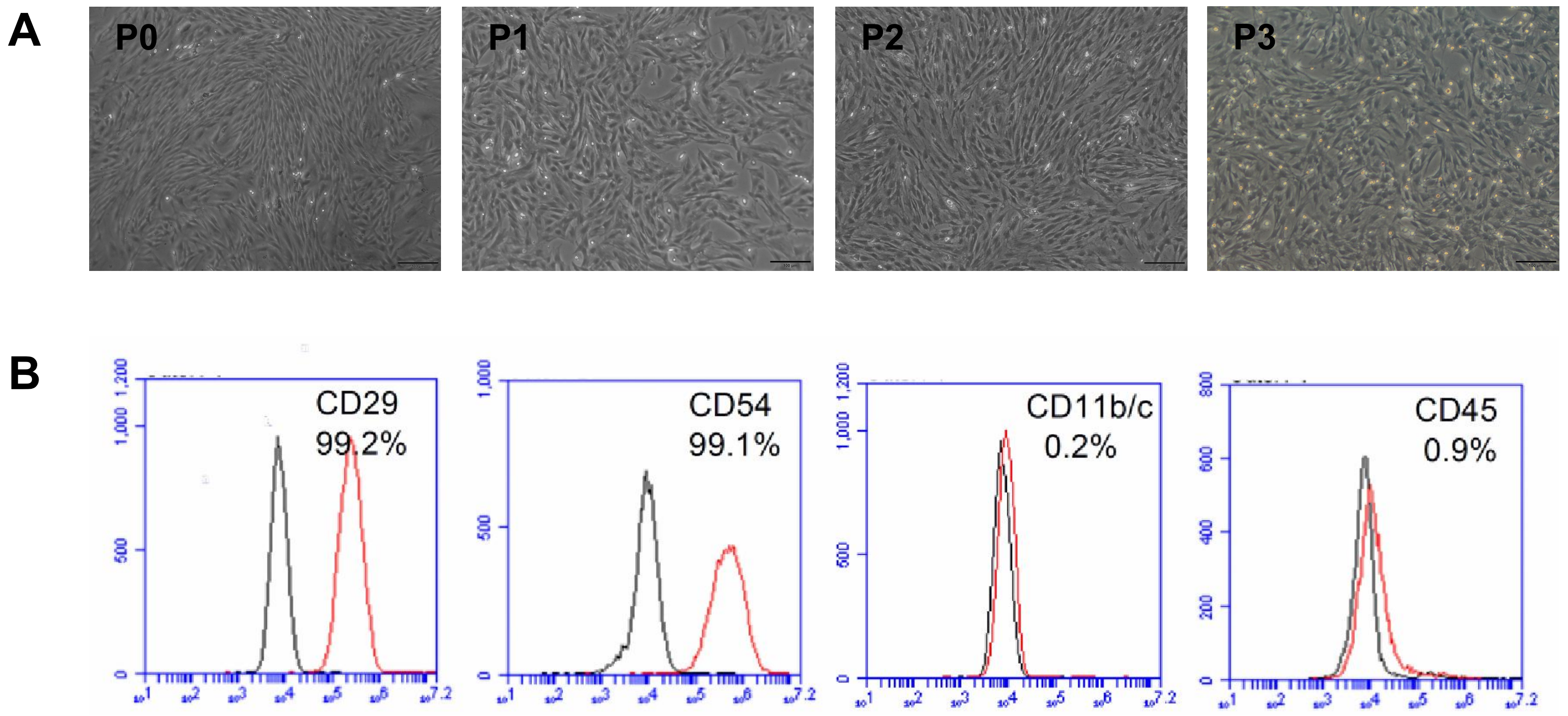

Figure S1. Identification of MSCs. (**A**) The P0-P3 MSCs showed a long spindle shape (bar=100 μm). (**B**) MSCs surface antigen identification by flow cytometry. MSCs: mesenchymal stem cells; Negative antigen: CD11b/c and CD45; positive antigen: CD29 and CD54.

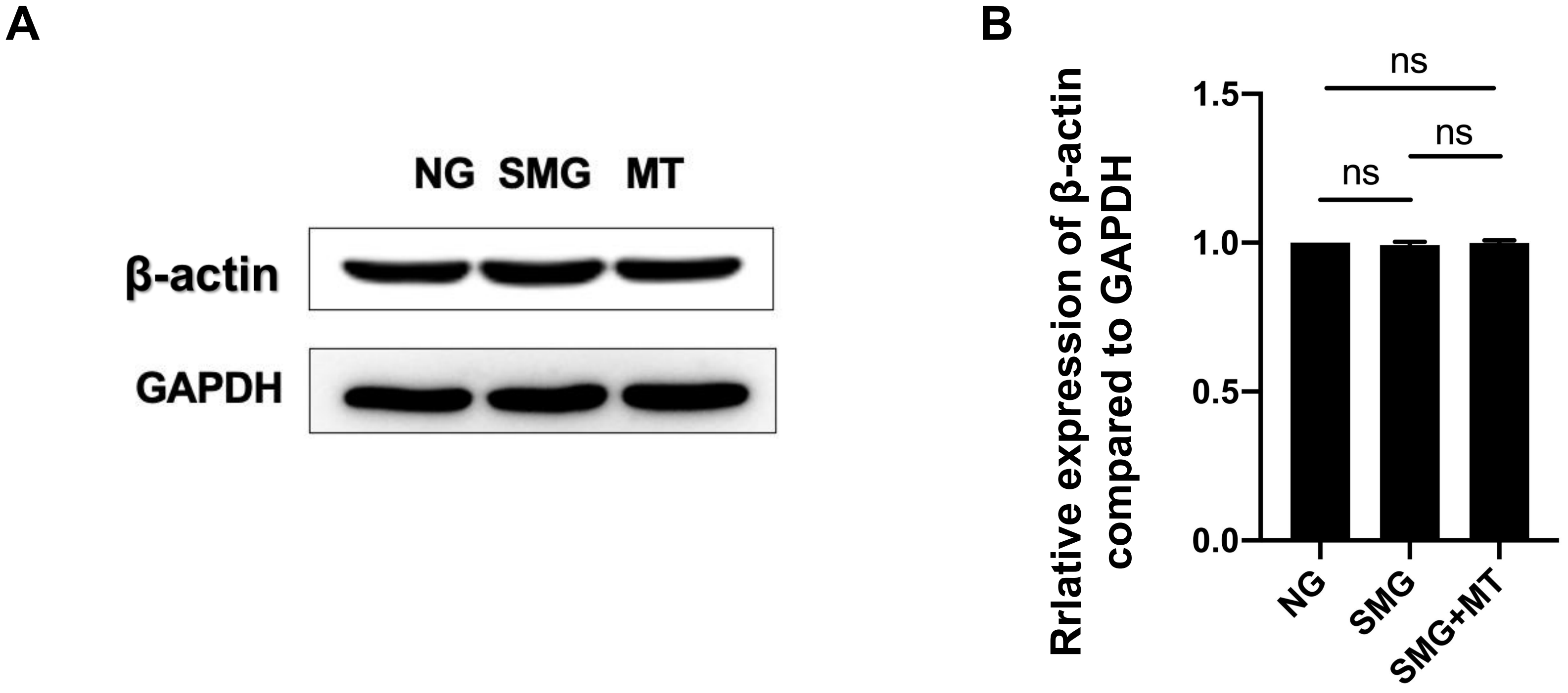

Figure S2. The representative image (**A**) and the quantitative analysis of the protein level expression of β-actin by normalizing to GADPH(**B**). MSCs: mesenchymal stem cells;NG: normal gravity; SMG: simulated microgravity; MT: Mito-TEMPO; n=3, ns $p>0.05$.
